# iPfam: a database of protein family and domain interactions found in the Protein Data Bank

**DOI:** 10.1093/nar/gkt1210

**Published:** 2013-11-30

**Authors:** Robert D. Finn, Benjamin L. Miller, Jody Clements, Alex Bateman

**Affiliations:** ^1^HHMI Janelia Farm Research Campus, 19700 Helix Drive, Ashburn VA 20147 USA and ^2^European Molecular Biology Laboratory, European Bioinformatics Institute (EMBL-EBI), Wellcome Trust Genome Campus, Hinxton, Cambridge CB10 1SD, UK

## Abstract

The database iPfam, available at http://ipfam.org, catalogues Pfam domain interactions based on known 3D structures that are found in the Protein Data Bank, providing interaction data at the molecular level. Previously, the iPfam domain–domain interaction data was integrated within the Pfam database and website, but it has now been migrated to a separate database. This allows for independent development, improving data access and giving clearer separation between the protein family and interactions datasets. In addition to domain–domain interactions, iPfam has been expanded to include interaction data for domain bound small molecule ligands. Functional annotations are provided from source databases, supplemented by the incorporation of Wikipedia articles where available. iPfam (version 1.0) contains >9500 domain–domain and 15 500 domain–ligand interactions. The new website provides access to this data in a variety of ways, including interactive visualizations of the interaction data.

## INTRODUCTION

Systems biology is the examination of the organization and dynamics of cellular and organismal function (1,2). Modern sequencing techniques have provided large sets of genes and proteins, with the complete, or near complete, repertoire of sequences known for many source organisms. However, to understand an organism at the system level, one requires knowledge of how these sets of proteins interoperate with other cellular components, such as other proteins, DNA or small molecular ligands, to achieve their function.

High-throughput experiments have provided large datasets of protein–protein interaction in a variety of organisms [e.g. (3–9)]. However, such experiments do not provide the molecular details that are often needed to understand function. Such data only comes from high-resolution 3D structures, but the number of known structures remains several orders of magnitude lower (<100 000), than the number of number of proteins (tens of millions). Therefore, it is important to develop methods that capture as much data as possible from the high-resolution data, thereby enabling the transfer of information and annotations to larger datasets [reviewed in (10)].

As well as protein–protein interactions, it is important to capture other types of interactions at a similar level of detail. The Protein Data Bank (PDB) (11) is the central repository of high-resolution 3D structures, and contains many protein bound small molecule ligands (simply referred to as ligand hereafter), some of which are important clinical drugs. Bound ligands can perform a functional role in the domain–ligand complex, such as the zinc ion that helps stabilize a zinc finger, while other ligands may actually disrupt function by (i) occluding protein binding sites (e.g. (12)), (ii) being analogous to metabolites (e.g. (13)) or (iii) binding to other functionally important regions (e.g. (14)).

To aid the detailed understanding of protein interactions, we have combined information from the Pfam database (which classifies protein sequence space into conserved evolutionary units) (15) and the PDB, to capture high resolution interaction data. This interaction data is made available in the database iPfam (16). iPfam was first released in 2004 (Pfam, release 12.0), as part of the Pfam database, providing detailed domain–domain interactions (DDI) data. In 2007, the Pfam web site underwent a complete redesign [described in (17)], aimed at improving Pfam data access and consistency across the Pfam mirror sites. Due to the exponentially larger sizes of both iPfam and Pfam datasets, Pfam was restricted to storing and displaying just DDI information, without residue, atom or bond information. Herein, we describe the first release of the standalone version of the iPfam database, which returns the details of domain interactions at residue and atomic resolutions.

iPfam has been expanded to include both DDI and domain–ligand interactions (DLI), whereas most similar, domain centric, resources exclusively provide either DLI (18–22) or DDI data (23–30). The new iPfam site provides easy access to the interaction data using a combination of a protein family (Pfam accession or identifier), a ligand and PDB identifier. We present visualizations for analyzing the interactions from both sequence and structure perspectives.

## METHODS

In the following section, we briefly describe the process of identifying domain interactions (both DDI and DLI). To identify Pfam protein family interactions in 3D structures, we have to take the Pfam data that is principally defined on UniProt sequences and transfer those annotations to the PDB atom coordinates, while taking account of the data in both resources being in flux.

### Mapping Pfam Entries to PDB Entries

The first step of the new, independent, iPfam update cycle is to synchronize the underlying databases, PDB and Pfam. iPfam version 1.0 is built using PDB entries as of 26 August 2013 and Pfam version 27.0, released in March 2013. Due to the asynchronous data release of the source databases, Pfam entries are mapped to PDB entries using a two-stage process.

The first stage depends on the high-quality Pfam–PDB mapping data provided by Pfam, which are constructed from a residue-to-residue mapping between PDB and UniProt called SIFTS (31). This approach of transferring annotations between sequence and structure using SIFTS has the advantage of being able to annotate the many protein fragments in the PDB, which are otherwise too short to significantly match a Pfam model. This allows the capture of many DDI what would otherwise look like a domain–peptide interaction. The Pfam–PDB mapping data is typically months out of date, so we process the mapping to remove references to obsolete PDB entries.

**Table 1.** Breakdown of the domain interactions that are available in iPfam version 1.0, according to the interaction type

	Homodomain	Heterodomain	Ligand
Intramolecular	528	2492	
Intermolecular	3993	5753	5168

The second stage addresses the fact that many new PDB entries will have been added since the Pfam mapping was generated and will, therefore, not be annotated with Pfam domains using the first stage. To make iPfam more comprehensive, we supplement the Pfam sourced Pfam–PDB mappings with data from RCSB PDB (32), which uses the HMMER web service (33) to annotate the PDB chain protein sequences with Pfam domains using the hmmscan search tool. This list of Pfam domain annotations is accessible via an API provided by RCSB PDB, and is used as a supplementary mapping data for structures not annotated by the first stage. Note, the RCSB PDB mapping may contain overlapping Pfam domains, the results are filtered to remove any overlaps, disregarding all but the most significant *E*-value match. Overall, this two-stage mapping approach resulted in 272 900 structural domains (from 6634 distinct Pfam entries) being identified in 87 386 different PDB entries.

### Defining Protein Family Interactions

Having performed the Pfam–PDB mapping, each PDB entry containing a Pfam protein family is analyzed for interactions. This is achieved by taking each domain and testing to see if any atoms in the domain are in close enough proximity to form a bond with any atom from another domain and/or a small molecule ligand found within the structure. We define the following bond types—covalent, electrostatic (or salt bridge), amino acid side chain and main chain (backbone) hydrogen bonds and van der Waals. All bonds are defined based on both the geometric (distance and angle) and chemical properties (charge, polarity and size) of the atoms involved, [as defined in (34), Chapter 4]. An interaction between a domain and another domain or ligand is called when one or more of these bonds are formed between the two molecules. Briefly, a covalent bond is determined if the distance between two atoms is less than the sum of the distance of the covalent radii of the atoms involved. An electrostatic bond is determined if the distance between the atoms is greater than the sum of the covalent radii and distance is less than the sum of the van der Waals radii, with one atom positively charged and the other negatively charged. A hydrogen bond is determined between two atoms that do not satisfy either the covalent or electrostatic criteria, and if one of the two atoms is a donor atom (D) and the other an acceptor atom (A). The D and A atoms must also satisfy the following geometric criteria: the D–A distance must be less than 3.6Å, and the angle between the vectors formed between D–A and A and amino acid residue atom, is between 90^o^ and 140^o^. A van der Waals bond is determined if the distance between the two atoms is greater than the sum of the atom’s van der Waal radii (*R*) and less than *R*+ 1.5 Å. The list of atom properties (charge, donor/acceptor, charge, covalent radii and van der Waals radii) used in the calculations is available on the iPfam FTP site (atomData.csv).

In iPfam, a small molecule ligand is defined as any molecule found in the PDB HETAM record, excluding nucleic acids, aqueous solvents and covalently modified residues. We use the 3-character alphanumeric code that the PDB assigns to each ligand in the Chemical Component Dictionary (http://www.wwpdb.org/ccd.html).

In the last release of iPfam, which was embedded in Pfam and contained the interaction data at the molecular level (release 20.0, April 2006), bonds arising from van der Waals interactions were stored as a property of the atoms forming that bond. Van der Waals bonds are general attractive forces that vary in strength based on the distance between two atoms. Thus, the close proximity of two amino residues tends to yield many van der Waals interactions. As a result, 96% of the 18 million recorded bonds were van der Waals. Since 2006, both the PDB and Pfam databases have grown substantially, and in an effort to make the production of iPfam more sustainable, the decision was taken to store van der Waals bonds as a property of the residues involved, rather than the actual atoms. Consequently, in iPfam 1.0, the protein-family atom-bond data table contains just 2.6 million records.

## iPfam STATISTICS

Overall, iPfam version 1.0 (referred to as 1.0 as it is the first release of the independent version of the database) catalogues 278 678 DDI structures, corresponding to 9516 distinct different domain pairings. Of these pairings, 43% (4074) are homo-domain interactions (i.e. both interacting domains belong to the same Pfam entry) and 57% (5442) correspond to hetero-domain interactions. Of the DDI, 6595 are inter-chain interactions (i.e. found on two separate chains/proteins in the PDB entry), 1354 are intra-chain and 1567 are found as both intra- and inter-chain. There are 529 800 structures of DLI, corresponding to 50 886 unique DLI pairings. Of the 15 540 ligands in the database, 90% (13 984) form an interaction with one or more Pfam entries. Many of the ligands not forming a DLI are typically interacting with a protein region not covered by a Pfam domain or with another molecule such as nucleic acids. Conversely, 78% (5168) of the families in Pfam with a known 3D structure bind one or more ligands. All of the interaction data contained in iPfam are available as a MySQL database dump available from the FTP site. Downloads of subsets of the data are possible via the web site.

## WEBSITE FEATURES

**Figure 1.**
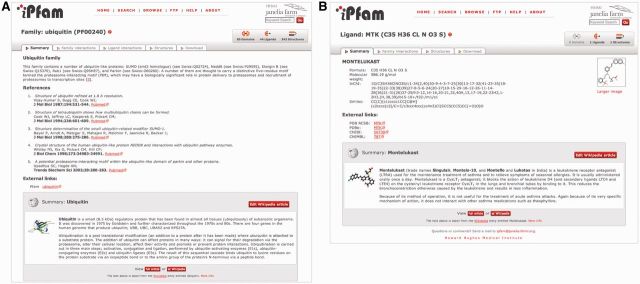
Examples of (**A**) the family and (**B**) the ligand pages showing the summary section or tab. Both pages present information about the respective entries and include external database links where possible. At the bottom of each page is the Wikipedia article summary.

The layout and design of iPfam share many similarities with the Pfam (15) and Dfam (35) web interfaces that we have developed. There are three main entry points to the iPfam data: a protein family, a ligand or a protein structure. At the top of every page is a navigation bar that provides links to the home page, browse pages, FTP site and help sections (Figure 1). Where appropriate in the site, we present an information box that summarizes the content of a section at a glance. This information box, if present, is located below the keyword search, on the top-right side of the page, and contains three icons indicating the number of DDI, number of DLI and number of structures cited in that section.

Interaction data may be retrieved by using the ‘jump to’ box on the homepage to navigate to the domain or ligand page of interest. These pages provide a coarse interaction detail, listing all domains and ligands that interact with the query where appropriate, as well as functional annotations. A page is typically broken down into a series of tabs that present the different data sections available. An example of the summary tab for both a protein family and ligand entry is shown in Figure 1A and B, respectively. In the summary tab for a Pfam family we display the one-line description of the entry and any associated annotation and references provided by Pfam. Beneath this text is a direct link to Pfam. Where possible we provide additional annotation from Wikipedia. The ligand summary tab contains more structured data that describes the ligand, including the ligand name, formula, molecular weight, InChI (IUPAC International Chemical Identifier) and SMILES (Simplified Molecular-Input Line-Entry System) string. For each ligand entry, external database cross-links are provided to both PDB RSCB and PDBe, and where possible to ChEBI (36) and ChEMBL (37). To provide further functional information about the 15 000 different ligands found in the PDB, we have started to link ligands stored in iPfam to the corresponding Wikipedia entries.

## FAMILY AND LIGAND WIKIPEDIA ARTICLES

We provide additional functional annotations for protein family entries by using the Wikipedia article mapping provided by Pfam. Linking between iPfam ligand entries and Wikipedia articles was performed by cross-referencing the stored ligand InChI with the InChI found in the Wikipedia chembox or drugbox infoboxes. Such infoboxes in Wikipedia chemical articles contain structured data that can be data-mined. iPfam currently maps to >2500 distinct Wikipedia articles (1484 family articles and 1110 ligand articles). The potential caveats of using Wikipedia data have been discussed previously, particularly with respect to the potential for vandalism (38). To try and minimize the risk of vandalism, we have built an in-house tool that captures snapshot summaries for each article. Each summary contains up to the first two paragraphs of the Wikipedia article lead section and an image, if available. The semi-automated summary generation is verified by a curator, who can modify (i) the length of the summary text (decreasing the number of sentences) and (ii) the selected summary image from the entire set of article images. Once the initial summary is prepared, the summary is stored in our database for use in the web site. Updated Wikipedia articles are retrieved on a daily basis and the summary is compared to the retrieved article to check for differences. Summaries where the corresponding text or images have been altered are flagged for review, with updates only being made after review. Currently, this results in about five summary reviews a day. This substantially reduces the likelihood of malicious vandalism being shown in iPfam by default. The full article can be revealed by selecting the ‘full article’ button or viewed and edited directly in Wikipedia. The use of summaries significantly reduces the amount of Wikipedia content that needs to be checked by a curator and provides a more uniform sized block of annotation to be displayed on the page.

## ACCESSING SPECIFIC INTERACTIONS

Adjacent to the family page summary tab, as shown in Figure 1A, are the family and ligand-interactions tabs. The table on each tab lists all detected interactions between the protein family (or domain) and other domains and ligands. These tables list the number of occurrences of each DDI, and additionally provide a breakdown of the occurrences according to whether the DDI is intrachain or interchain. The user can access details of the pair-wise interaction by using the ‘Structures’ and ‘Sequences’ buttons in the table. Selecting either of these buttons takes the user to the next level of detail in the site, altering the context in which the data will be presented. For example, from the ubiquitin family interaction tab (http://ipfam.org/family/PF00240/fam_int), the user can enter the ‘ubuiquitin–CUE interaction’ section of the site to investigate the interaction between the two domains. When using the ‘structure’ button, users are taken to a list of structures containing the ubiquitin–CUE interaction, which tabulates each structural example of the DDI and includes a summary of the bonds that form the interaction. From here, the user can explore each example of the interaction in even more detail. Alternatively, using the ‘sequence’ button from the top-level family interaction tab, the user is taken directly to the sequence interaction graphical display (described below). Similarly, the ligand interaction tab lists ubiquitin interactions with different small molecule ligands, which can be explored in the same fashion as the DDI. The final tab on the family page is the download tab, which provides tab-delimited versions of the data contained in the interaction data tables found in the family page tabs. The top-level ligand pages have a layout similar to the family pages.

## VISUALIZING INTERACTIONS

The interaction data contained within iPfam may be investigated at both the residue and the atomic level, using 2D and 3D visualizations. The user may invoke these visualizations by descending through the site as described above, or simply by searching with a PDB identifier.

### 2D interaction visualization

**Figure 2.**
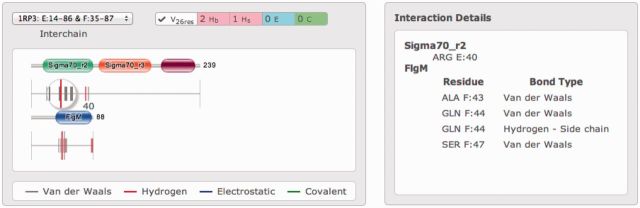
2D representation of a DDI between Sigam70_r2 and FlgM, as determined from the structure 1SC5. The alternative class σ-factor, σ^28^ is composed of three separate domains [colored green (Sigma70_r2, PF04542), orange (Sigma70_r3, PF04539) and dark red (Sigma70_r4, PF04545)], named after the conserved regions in the primary housekeeping transcription factor, σ^70^ [(39) and references therein]. In this case, residue 40 of the Sigma70_r2 domain has been selected, with the interacting residues in FlgM (colored blue, Pfam accession: PF04316) being displayed by making the colored vertical lines large. The details of the bonds are also shown in the adjacent ‘Interaction Details’ display.

Many structures in the PDB contain multiple domains, with multiple interactions between the domains. For example, the structure of *Aquifex aeolicus* transcription factor σ^28^, bound by the anti-σ FlgM [PDB identifier 1SC5, (39)] provides us with the molecular details of how these two proteins interact. The interaction interface is extensive yet discontinuous, with FlgM binding occluding both the core RNA polymerase-binding interface and the DNA-binding sites of the σ-factor. However, this complex interaction can be simplified as a set of 2D displays for each DDI, as shown in Figure 2. This display graphically represents the domain organization of each chain, with the positions of interacting residues indicated on a line below each domain organization graphic. Each vertical bar on this line indicates the position of a residue that is involved in forming the interaction interface, and is colored according to the most energetically significant bond found at that position. Placing the mouse cursor over the vertical bar causes the interacting residues in the other sequence to be highlighted, by increasing the line height. The details of the interaction(s) between the subject residue and residue(s) from the other domain are displayed in a box to the right of the graphic (as shown in Figure 2). Similarly, with DLIs the sequence containing the interacting domain is represented graphically, with bond-forming residues shown below. The ligand is not represented in this view, with the interactions between domain residues and ligand listed in the ‘Interaction Details’ box. All of the residue interaction details may be downloaded using the adjacent download tab.

### 3D visualization interface

The atomic resolution interaction data in iPfam is the most fine-grained data, and is best visualized using the 3D structure that it is calculated from. One of the early design decisions made with the iPfam web site and structure visualization was to adopt the JavaScript version of Jmol (40), JSmol, in preference to the applet version. Applets require Java to be installed locally and to be correctly configured within the browser, whereas JSmol uses the browser to render the structure. The drawback of JSmol is that it uses HTML5 and canvas, which is not supported by older browsers, particularly Internet Explorer version 8.0 and earlier. Given that these older browsers make up <10% of the likely usage (and this fraction will diminish over time), we felt that supporting only JSmol was the appropriate decision given limited development resources. As outlined below, using the JavaScript version has allowed a more elaborate integration of data and structure visualization.

**Figure 3.**
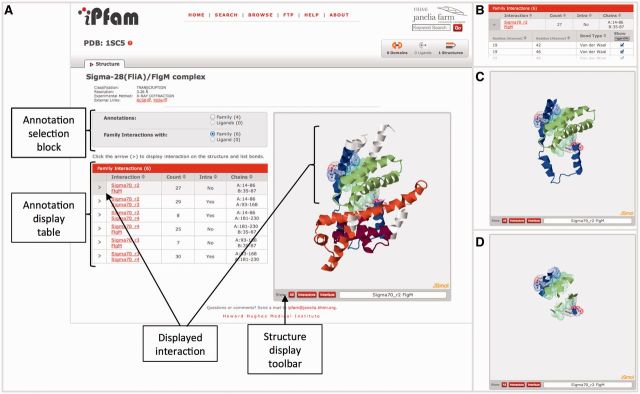
Structure of the *Aquifex aeolicus* transcription factor σ^28^, bound by the anti-σ FlgM (PDB identifier 1SC5). (**A**) The interaction between σ^28^ and FlgM is multipartite, with FlgM wrapping around the σ-factor, preventing normal function. The Pfam domains in the structure are colored as in Figure 2. The interaction between FlgM and Sigma70_r2 has been selected from the annotation display table, causing the interaction to be displayed in the structure. (**B**) A partial view of the nested table listing all of the interactions bonds between the selected domains. The ‘Toggle VDW’ button in the header and checkboxes can be used to modulate which bonds are displayed in the structure. (**C**) The JSmol structure view in (A) has been modified to show just the interacting domains, using the ‘Interactors’ button from the toolbar. (**D**) The structural display has been further restricted to show just the interacting residues using the ‘Interface’ button.

The iPfam structure page offers visualization of all structures that were available at the time of the release, whether an interaction had been found or not. For each structure, the protein chain or chains are rendered as white ribbon cartoons, depicting the secondary structure elements. The positions of Pfam domains are indicated on the structure by coloring of the ribbon, with each domain in the structure assigned a unique color. Ligands, if present, are shown as a ball-and-stick representation and colored according to the CPK scheme. Figure 3 shows an example of a structure page using the PDB entry 1SC5 (39).

Factors such as the size, the number of the domains and the presence of multiple copies of the same domain all complicate visualizing a domain of interest in a structure. To simplify identification of each domain in a structure, the user can switch to ‘Family’ annotations using the radio button found in the annotation selection block (Figure 3A), which causes the lists of the Pfam domains in the structure to be shown in the annotation table below. Placing the mouse cursor over the domain of interest in the list of interactions provided in the annotation display table causes the corresponding domain in the structure to be highlighted. Similar highlighting is also available for ligands, but rather than changing the color, enlarges the ball and stick representation of the ligand, making it easy to locate.

To view the details of an interaction, the user first chooses the desired interaction annotation (DDI or DLI) using the radio buttons in the annotation selection block, followed by activating the display of the specific annotation by clicking on the ‘>’ symbol in the table of annotations, as shown in Figure 3A. This click triggers the following set of events: (i) reveals the list of bonds (hidden by a subsequent click in Figure 3A, but shown in Figure 3B), (ii) renders the bonds on the structure and (iii) activates the tool bar underneath the JSmol pane. This toolbar contains three buttons that control how much structure information is displayed. The ‘All’ button shows the entire structure, Figure 3A. To provide a less obscured investigation of each domain interaction, it is possible to hide the parts of the structure that do not belong to the interacting domains by clicking on the ‘Interactors’ buttons, Figure 3C. The ‘Interface’ button restricts the display still further, so that just the interacting residues are visible, Figure 3D. In all three views, the nested table showing the list of bonds can be used to modulate the display of each bond, by clicking on the checkboxes (Figure 3B). All van der Waals bonds can be toggled off using the button in the table header (labeled ‘Toggle VDW’, Figure 3B), leaving just the higher order bonds visible.

**Figure 4.**
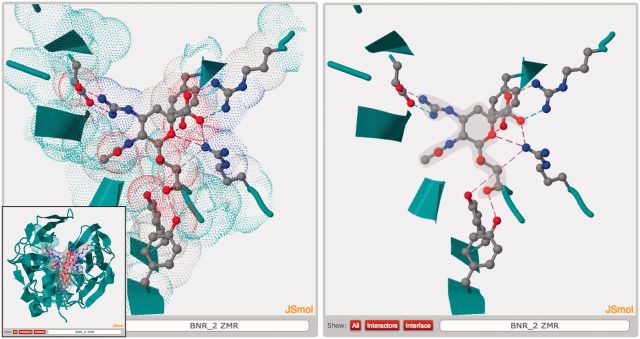
The binding interface between the neuraminidase inhibitor, Zanamivir and the Human sialidase Neu2 containing the BNR_2 (PF13088) domain (PDB identifier 2F0Z). The complete DLI is shown in the insert, bottom left. The left panel shows the interface between the domain and ligand. The ligand is rendered as a ball-and-stick representation. Interacting amino acids from BNR_2 domain are rendered using the cartoon representation, with interacting amino acid side chains as CPK colored, ball-and-sticks. Residues that form van der Waals interactions are drawn with their surrounding van der Waals radii indicated by a dotted halo around the entire amino acid. These van der Waals may be toggled off (see Figure 3B), to display just the higher order bonds, as shown in the right panel. For illustrative purposes only, the Zanamivir ligand is shown with a semi-transparent grey overly. The blue, dashed lines between the ligand and side chain indicate the positions of the electrostatic bonds. The dashed pink lines similarly indicate the positions of hydrogen bonds.

Figure 4 illustrates how an interaction interface is rendered in structure, using the DLI between Zanamivir, a drug, and the Human sialidase Neu2 protein (PDB identifier 2F0Z, unpublished). Numerous interactions are made between the BNR_2 domain (PF13088), and the ligand within the cleft of the domain (insert in Figure 4). The two panels in Figure 4 show just the interaction interface, using the toolbar to restrict the display as described above. The ligand, Zanamivir, is shown as a ball-and-stick and highlighted in the right panel by a semi-transparent overlay on the figure. Using the display controls provided in the page, the rendering can be quickly modified to enable the visualization of just the higher order bonds (dashed lines), in this case between the amino acid side chains and the bound molecule.

## DISCOVERING INTERACTION DATA

In addition to the browse pages and the ‘jump to’ sections found on the front page, we provide two additional search interfaces to the database. The first is via a keyword search that matches a query string against the textual data associated with the Pfam families, ligands, PDB entries and linked Wikipedia articles. The second search interface allows iPfam to be queried via a protein sequence.

**Figure 5.**
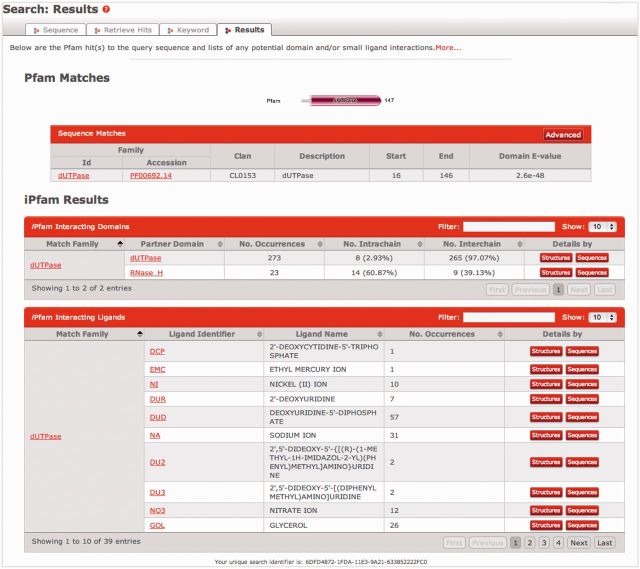
The results from searching iPfam with the Deoxyuridine 5′-triphosphate nucleotidohydrolase from yeast (UniProt accession P33317). The top section shows the Pfam match to the dUTPase model (PF00692). Below this is a list of known DDIs and DLIs found in iPfam for dUTPase. The text field located in the header provides a simple way of filtering through the results.

The protein sequence search tool does not predict interactions in the sense that it does not compare the query sequence to the sequences from the structures that were used to determine the interaction. Instead, this provides a convenient means of querying for a set of interactions based on the Pfam domains found on the query sequence. To perform this search, Pfam domains are identified in the query sequence using the HMMER web service (33). The iPfam server then performs a lookup of the previously observed DDI or DLI for each identified domain. Both the Pfam and iPfam results are returned to the browser and displayed as shown in Figure 5. The top section of the results, Figure 5, shows the position of the domains in both graphical and tabular forms. Under the Pfam results are the iPfam interactions for the domains found on the query sequence. As the lists of interacting partners are potentially very large, we have included the ability to filter the results using a simple text-filtering tool, which is applied to all table cells. Such a search can provide a platform for exploring the interaction data in iPfam in more detail and be used to guide docking experiments.

## FUTURE DIRECTIONS

The separation of iPfam and Pfam not only facilitates the independent development and updating of the two resources, but also allows a clearer segregation between protein family sequence annotations and domain interaction annotations. Both databases will develop data exchange mechanisms that will allow the display of the interaction data in the context of family multiple sequence alignments. This will allow interfaces to be compared and analyzed with respect to family sequence conservation. As part of the ongoing developments in iPfam, we will investigate the inclusion of domain–nucleic acid interactions, another important class of interaction not currently represented in the database, to provide a comprehensive and detailed set of protein interaction annotations.

A source of both false-positive and false-negative interactions in iPfam is that the interactions are currently calculated only on the asymmetric unit found in the PDB file. These may or may not reflect the true biological assembly of the protein and may contain crystal-packing artifacts (41). Having re-established iPfam, we will be investigating the use of the biological rotation matrices supplied in the PDB files, to provide additional arrangements of the proteins and hence interactions. One way of minimizing the number of crystal contact artifacts, or false-positive interactions, is to look at the properties of the interaction interface (42–46).

We do not currently impose any threshold criteria on the number of bonds or the amount of surface area that needs to be found at an interface, because true DDI interfaces can be very small. For example, in the Human BCR-ABL tyrosine kinase the SH3 domain interacts with a small region of the SH2 domain that is essential for orienting peptide interactions [PDB identifier 2ABL, (47)]. This interface consists entirely of van der Waals bonds, formed between just five residues from the SH3 domain and four residues from the SH2 domain (interface size of ∼125 Å). Furthermore, as we treat each structural DDI in isolation, a small, weak, yet biologically meaningful interaction may occur between two interchain domains, which would on in its own be insufficient to pass any criteria. However, a second more substantial interchain interaction may be mediated by another DDI, primarily facilitating the interaction between the two chains.

We acknowledge that the lack of an interface threshold could be a source of some false-positive interactions, and we will incorporate interface area metrics in the future to provide users with as much discriminatory information as possible about the interface.

We also do not try to discriminate between different small molecules in the DLI calculations. The presence of a co-crystallized ligand may be functionally important or simply an artifact due to its presence in the crystallization buffer. The aim of the iPfam database is to harvest as much of the high-resolution interaction information as possible from the few known structures, and to present this data in an accessible way that allows the user to make a judgment regarding the biological relevance of an interaction.

## CONCLUSION

The iPfam database is constructed from two continuously updating sources, PDB and Pfam, both of which are well established in their respective fields of 3D structure data and protein domain data. The simple, rich user interface in iPfam, combined with the popularity of the source databases, makes interaction data available to a wide audience that includes both bench biologists, looking at their favorite sequence, and computational biologists, requiring comprehensive interaction data for their own data mining purposes. Bringing both DDI and DLI interaction data into the context of protein families, via the unified framework in iPfam, will help researchers to better understand the relationship between ligand and domain binding at a molecular level. There is an increasing body of evidence showing that DDI and DLI interfaces often overlap, allowing the knowledge of one class of interaction to be used to derive hypotheses about both classes of interaction (48–50). Combining knowledge of protein families, large-scale protein-interaction datasets and detailed molecular interaction data will not only improve our understand of biological systems, but also forms an important basis for the rational design of drugs that can modulate the constituent pathways (49,50,51).
